# Systems Immunology of Diabetes-Tuberculosis Comorbidity Reveals Signatures of Disease Complications

**DOI:** 10.1038/s41598-017-01767-4

**Published:** 2017-05-17

**Authors:** Cesar A. Prada-Medina, Kiyoshi F. Fukutani, Nathella Pavan Kumar, Leonardo Gil-Santana, Subash Babu, Flávio Lichtenstein, Kim West, Shanmugam Sivakumar, Pradeep A. Menon, Vijay Viswanathan, Bruno B. Andrade, Helder I. Nakaya, Hardy Kornfeld

**Affiliations:** 1 Department of Pathophysiology and Toxicology, School of Pharmaceutical Sciences, University of São Paulo, 05508 São Paulo, Brazil; 2 Laboratório de Imunoparasitologia, Instituto Gonçalo Moniz, Fundação Oswaldo Cruz, Salvador, Brazil; 3 National Institutes of Health- NIRT - International Center for Excellence in Research, Chennai, India; 4 Unidade de Medicina Investigativa, Laboratório Integrado de Microbiologia e Imunorregulação, Instituto Gonçalo Moniz, Fundação Oswaldo Cruz, Salvador, Brazil; 5 Multinational Organization Network Sponsoring Translational and Epidemiological Research, Instituto Brasileiro para a Investigação da Tuberculose, Fundação José Silveira, Salvador, Brazil; 6 Curso de Medicina, Faculdade de Tecnologia e Ciências, Salvador, Brazil; 7 Department of Medicine, University of Massachusetts Medical School, Worcester, Massachusetts United States of America; 8 National Institute for Research in Tuberculosis, Chennai, India; 9 Prof. M. Viswanathan Diabetes Research Center, Chennai, India; 10 Universidade Salvador (UNIFACS), Laureate Universities, Salvador, Brazil; 11 Division of Infectious Diseases, Department of Medicine, Vanderbilt University School of Medicine, Nashville, USA

## Abstract

Comorbid diabetes mellitus (DM) increases tuberculosis (TB) risk and adverse outcomes but the pathological interactions between DM and TB remain incompletely understood. We performed an integrative analysis of whole blood gene expression and plasma analytes, comparing South Indian TB patients with and without DM to diabetic and non-diabetic controls without TB. Luminex assay of plasma cytokines and growth factors delineated a distinct biosignature in comorbid TBDM in this cohort. Transcriptional profiling revealed elements in common with published TB signatures from cohorts that excluded DM. Neutrophil count correlated with the molecular degree of perturbation, especially in TBDM patients. Body mass index and HDL cholesterol were negatively correlated with molecular degree of perturbation. Diabetic complication pathways including several pathways linked to epigenetic reprogramming were activated in TBDM above levels observed with DM alone. Our data provide a rationale for trials of host-directed therapies in TBDM, targeting neutrophilic inflammation and diabetic complication pathways to address the greater morbidity and mortality associated with this increasingly prevalent dual burden of communicable and non-communicable diseases.

## Introduction

The adverse effect of type 2 DM on TB severity at presentation, treatment response, outcomes and relapse has become a major global public health concern due to rapidly increasing DM prevalence in countries where TB prevalence was already high^1^. The mechanisms responsible for TB susceptibility and severity in DM are not well understood and there is a paucity of data to inform the management of TBDM patients and the design of interventional and implementation trials to address this problem. Susceptibility of diabetic mice results from a delayed innate immune response to the alveolar macrophages initially infected with inhaled *Mycobacterium tuberculosis*, resulting in several additional days of logarithmic bacterial replication^2, 3^. Once expressed in the lung, T cell mediated immunity is qualitatively intact but with a quantitatively greater burden of inflammatory pathology due to the higher plateau bacterial burden along with possible defects in counterregulation^4^.

Investigating early events in human TB is challenging since patients typically present more than 2 months after disease onset^5^. Furthermore, comorbid DM specifically increases the risk for pulmonary more than extrapulmonary TB^6^ which presents another barrier to clinical investigation since sampling the site of disease is impossible in most settings. The blood transcriptome offers a reflection of immunological events in the lung and a consensus gene expression signature of active TB is emerging from observational studies in nondiabetic TB patients in Africa, China, Europe and Indonesia^7^. To gain insights into mechanisms of TB susceptibility in human DM and to assess the impact of TB disease on diabetic complications, we leveraged data and blood samples from diabetic and nondiabetic TB patients enrolled in the Effects of Diabetes on Tuberculosis Severity (EDOTS) study in Chennai, India^8^. Integrative analysis applied to these data showed a high degree of comparability in the blood transcriptional response to TB between diabetic and nondiabetic participants but a distinct signature of plasma cytokine and growth factor levels and an association of TBDM with neutrophilic inflammation. The data further show that comorbid TB activates a range of pathways associated with diabetic complications, above the levels observed with DM alone. We conclude that neutrophilic inflammation and diabetic complication pathways may be useful targets for host-directed therapies to improve TB treatment and outcomes in this growing patient population.

## Results

### Study population

EDOTS is a longitudinal, observational cohort study comparing adult participants with pulmonary TB in Chennai, India, who are rigorously classified as diabetic or normoglycemic according to WHO guidelines^8^. The investigations reported here used demographic and clinical data as well as stored samples of plasma and whole blood RNA collected at enrollment from 60 EDOTS cohort participants (30 diabetic and 30 normoglycemic). Since the phenotype of TBDM might differ between participants who were diabetic prior to incident TB and those with an initial diagnosis of DM at the time of enrollment in EDOTs, we restricted the current investigation to participants with preexisting DM. Retreatment of TB, prior active TB diagnosis and HIV infection are exclusion criteria for EDOTS. TB diagnosis was classified by positive sputum culture for *M*. *tuberculosis*. Comparison was made with 60 control participants without active TB disease, of whom 30 were classified as diabetic and 30 as normoglycemic using identical case definitions. Demographic and behavioral differences were identified between participants with TBDM dual burden, TB alone, DM alone and healthy controls (Supplemental Table 1). Those with TBDM tended to be older than normoglycemic participants with TB but less likely to report tobacco or alcohol consumption. The healthy control and DM subgroups had comparable, normal median body mass index (BMI) whereas median BMI was significantly lower in the TBDM and TB subgroups.

### Demographic, behavioral and clinical laboratory parameters

**Figure 1:**
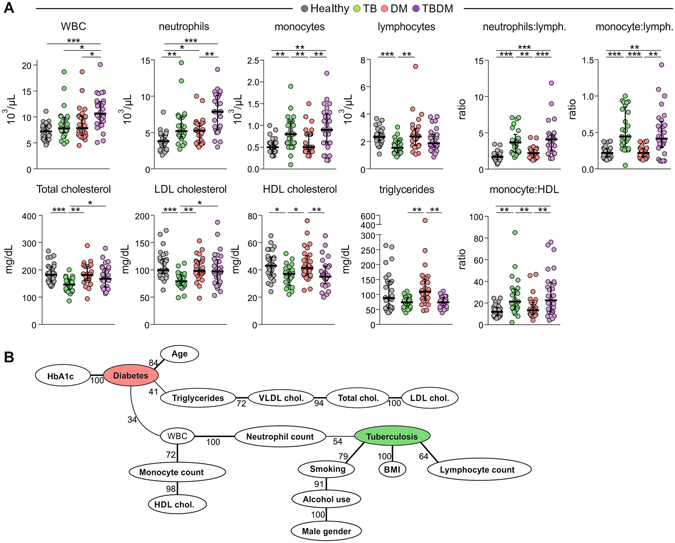
Factors associated with TB and/or DM in the cohort. (**A**) Data represent median and interquartile ranges. The Kurskal Wallis test with Dunn’s multiple comparisons ad hoc test were employed to compare the values detected between the study subgroups. *P*-values for the Kruskal Wallis tests are shown in Appendix File S1. *P*-values were adjusted for multiple comparisons using Holm-Bonferroni’s approach as described in methods. Only comparisons with significant *P*-values after adjustments are displayed (*P < 0.05, **P < 0.01, ***P < 0.001). (**B**) Bayesian network with bootstrap (100x) was used to illustrate the statistically significant associations between the parameters and the presence of TB and/or DM in the study population. Lines represent direct associations. Associations that remained statistically significant on >30 times out of 100 bootstraps are plotted. Numbers of times each association persisted during bootstrap are shown. Bold lines highlight the strongest associations, which persisted more than 60 times in the bootstrap. Vitamin D is not presented in the picture, as it would be solitary node with no arcs attached.

Among the 120 participants in the current investigation, median glycohemoglobin (HbA1c) was 5.5%, 7.5%, 5.7% and 10.2% in the subgroups of healthy controls, DM, TB, and TBDM, respectively (Supplemental Table 2). All subgroups were vitamin D insufficient (<20 ng/mL; Supplemental Table 2)^9^. Median circulating leukocytes and absolute neutrophil count were higher in TBDM patients than the other subgroups, but both DM subgroups had higher neutrophil counts than healthy controls (Fig. 1A and Supplemental Table 2). The neutrophil:lymphocyte ratio (NLR), a biomarker associated with TB severity^10^, insulin resistance and type 2 DM^11, 12^ and coronary artery disease risk^13, 14^ was higher in the TB and TBDM subgroups compared to DM and to healthy controls. Participants with TBDM had lower serum triglycerides and lower HDL than the DM subgroup (Fig. 1A). The monocyte:HDL ratio (MHR), a biomarker associated with coronary artery disease risk and severity^15^ was also higher in the TBDM and TB subgroups compared to DM and to healthy controls.

We applied Bayesian Network modeling to infer causal relations between the presence of TB and/or DM and the recorded demographic, behavioral and clinical laboratory parameters (Fig. 1B). The network learned from these variables represents the minimum probabilistic dependency between the variables that explains most of the observed correlations in the data^16^. This confirmed the expected associations of DM with patient age, HbA1c, triglycerides and VLDL, total and LDL cholesterol. Gender (male), alcohol use and smoking formed an association chain with TB, while BMI and lymphocyte count were also strongly associated with TB. Neutrophil count was identified at the nexus between DM and TB. Altogether, these data indicate that neutrophilic inflammation is a central feature of TBDM and that incident TB disease elevates levels of biomarkers associated with diabetic vascular complications above the levels found in DM without comorbid TB.

### Signature pattern of elevated plasma cytokines and growth factors in TBDM comorbidity

**Figure 2:**
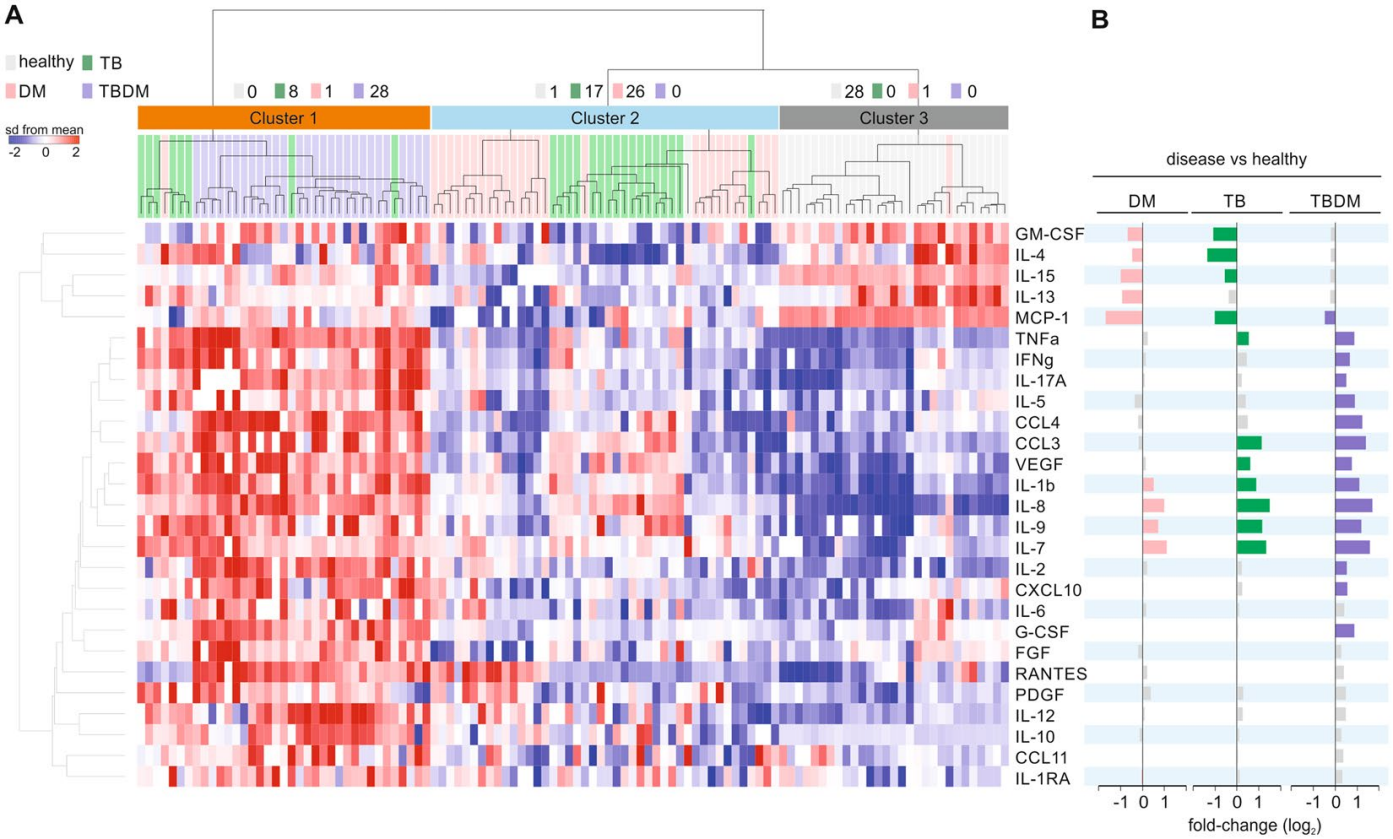
Cytokine profiling in plasma of patients with TB and/or DM. (**A**) Cytokine concentrations were z-score normalized across all subjects. Each column represents one patient. The cytokine names are shown to the right of the heat map. Cytokine profiles were ordered by hierarchical clustering (Euclidean distance and clustered with ward method). The condition tree at the top shows 3 main clusters. The number of subjects from each clinical phenotype (class) on each cluster is indicated. (**B**) Differences in cytokine levels for each disease compared to healthy subjects. Differences which did not reach statistical significance (Adjusted *P* < 0.05, fold-change >1.4) are represented as grey bars.

Elevated plasma levels of a broad range of cytokines in TBDM patients have been reported in diverse populations^17–19^. To assess this relationship in our cohort of TB patients and controls, we measured plasma levels of 27 cytokines and growth factors by Luminex immunoassay (Fig. 2 and Supplemental Table 3). Primary analyses revealed that several analytes exhibited statistically different concentrations in plasma between the study groups (Supplemental File 1). Data was then z-score normalized across the entire cohort and analyzed by hierarchical clustering. Three main clusters were identified and showed 100% separation for TBDM vs. healthy controls, with more overlap between the TB and the DM subgroups. The cluster containing all TBDM cases (and 32% of TB cases) was notable for higher expression of nearly all analytes tested (Fig. 2A and Supplemental File 1). Surprisingly, another cluster contained both the majority of nondiabetic TB cases (68%) and the majority of diabetic controls without TB (93%). Growth factors elevated in TBDM included VEGF, PDGF and FGF that are all implicated in diabetic complications^20–22^ (Supplemental File 1). Compared to healthy subjects, the levels of several analytes (IFNg, IL-17A, IL-5, CCL4, IL-2, CXCL10, and G-CSF) were significantly higher only in the TBDM subgroup (Fig. 2B). We conclude that a signature pattern of plasma levels of cytokines and growth factors distinguished TBDM from DM and healthy control patients, and from a majority of nondiabetic TB patients in this cohort.

### Blood transcriptome profile of patients with TB and/or diabetes

**Figure 3:**
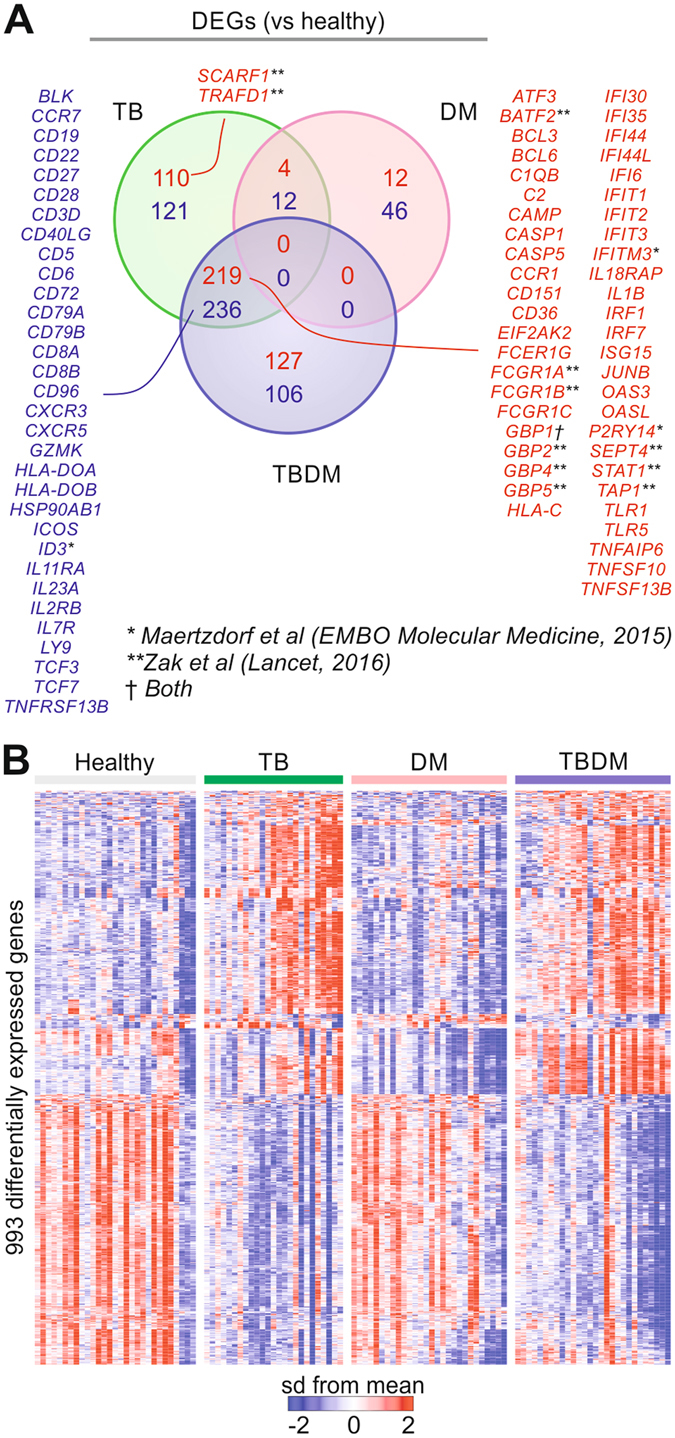
Transcriptomic changes with TB and/or diabetes compared to healthy subjects. (**A**) Differentially expressed genes (DEGs) in patients with TB and/or DM compared to healthy subjects (adjusted *P* < 0.05, fold-change >1.4). The Venn diagram shows the number of DEGs in common to two or more clinical phenotypes or unique to only one disease. (**B**) Expression patterns of the DEGs from (**A**). Cohort subgroups are shown by the colored bars. Each column represents one subject. The genes (rows) were normalized by z-score across all samples.

To investigate the impact of DM on human immunity to TB we performed whole blood gene expression profiling of all participants in the cohort. A total of 993 differentially expressed genes (DEGs) were identified, comparing the TBDM, TB and DM subgroups to the healthy subjects (Fig. 3A, Supplemental File 2). The expression patterns of these DEGs across all samples revealed considerable heterogeneity among individuals within each subgroup (Fig. 3B). Most of the DEGs (935) were identified comparing TB or TBDM classes vs. healthy controls. Among these, 455 genes were commonly found in TB and TBDM comparisons. These included genes found in previously reported TB signatures^23, 24^ (Fig. 3A). Only 74 DEGs were found between DM patients and healthy subjects (Fig. 3A). We conclude that the immune response to *M*. *tuberculosis* infection is the major driver of the blood transcriptomic changes seen in TBDM patients and this response is only mildly perturbed by DM as compared to the response in nondiabetic individuals with pulmonary TB disease.

**Figure 4:**
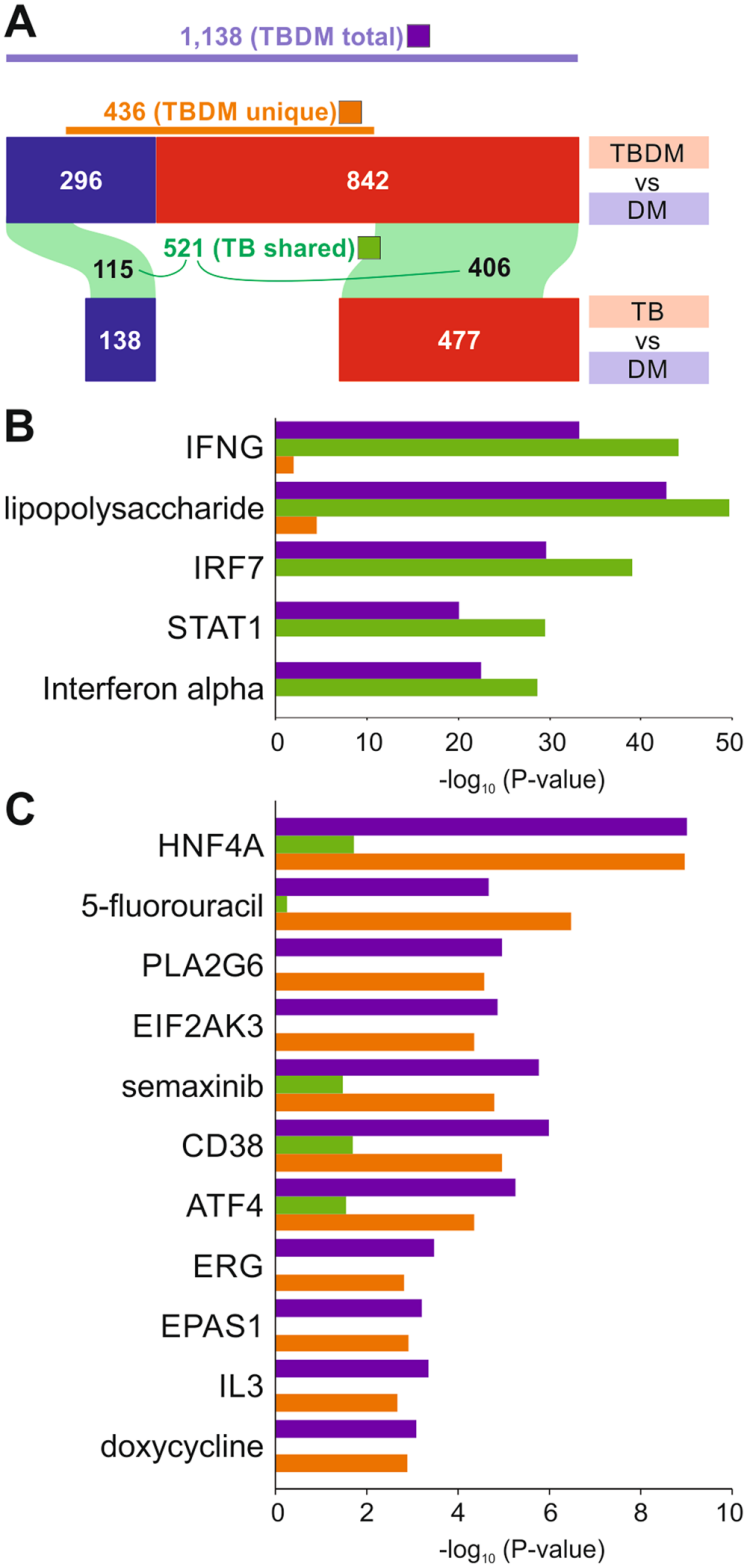
Blood transcriptome of patients with dual TB and DM burden compared to TB or DM alone. (**A**) Differentially expressed genes between patients with distinct clinical phenotypes (class). The comparison between two classes is indicated at the top of each bar. Red and blue bars represent the number of DEGs which are up- or down-regulated, respectively in the first class of the comparison. The purple bar shows the number of DEGs in the comparison TBDM vs. DM (“TBDM total”). The number of DEGs which are in common to two comparisons are shown inside green areas connecting two horizontal bars (“TB shared”). Genes which are uniquely differentially expressed in TBDM vs. DM is shown by the brown bar (“TBDM unique”). (**B**) Top transcriptional regulators in “TB shared” DEGs. Ingenuity’s Upstream Regulator Analysis was performed using “TB shared” (green bars), “TBDM unique” (brown bars), and “TBDM total” DEGs (purple bars). The x-axis indicates the significance of the enrichment for the upstream regulators on the left. (**C**) Top transcriptional regulators in “TBDM unique” DEGs identified by the same approach as in (**B**).

We next compared differences in the transcriptome profiles between participants with TBDM and participants with either TB or DM alone (Fig. 4A, Supplemental File 2). Whereas little change was found between the TBDM and TB subgroups (62 DEGs), a total of 1,138 genes were differentially expressed in TBDM compared to DM patients (“TBDM total”). Of those, 521 genes were also differentially expressed in in the comparison between TB patients and DM patients (“TB shared”). The remaining 436 genes were uniquely differentially expressed in the TBDM vs. DM comparison, reflecting an impact of TBDM comorbidity on blood gene expression distinct from the influence of either TB or DM alone (“TBDM unique”). Ingenuity Pathway Analysis applied to these gene lists revealed the main possible transcriptional upstream regulators driving the TB (Fig. 4B) and TBDM (Fig. 4C) responses. As expected, TB disease was associated with the induction of genes related to types I and II IFN responses (Fig. 4B). Among potential regulators significantly enriched in “TBDM unique” genes, several were related to endoplasmic reticulum (ER) stress, including HNF4A, ATF4, EIF2AK3, and PLA2G6. Glucotoxicity and lipotoxicity are known to promote ER stress in type 2 DM^25^ but our results indicate that comorbid TB further exacerbates ER stress in diabetic hosts. This may be clinically significant since ER stress is linked to loss of β-cell mass in type 2 DM and is implicated in diabetic complications^25, 26^. The “TBDM unique” genes were also significantly enriched by targets of the anti-cancer drugs 5-fluorouracil and semaxanib, and the antibiotic doxycycline (Fig. 4C). These three different classes of drugs share a common link with the DNA damage response pathway^27–29^, which is associated with insulin resistance and microvascular complications of DM^30^.

### Molecular degree of perturbation is elevated in TB and diabetes alone and in combination

**Figure 5:**
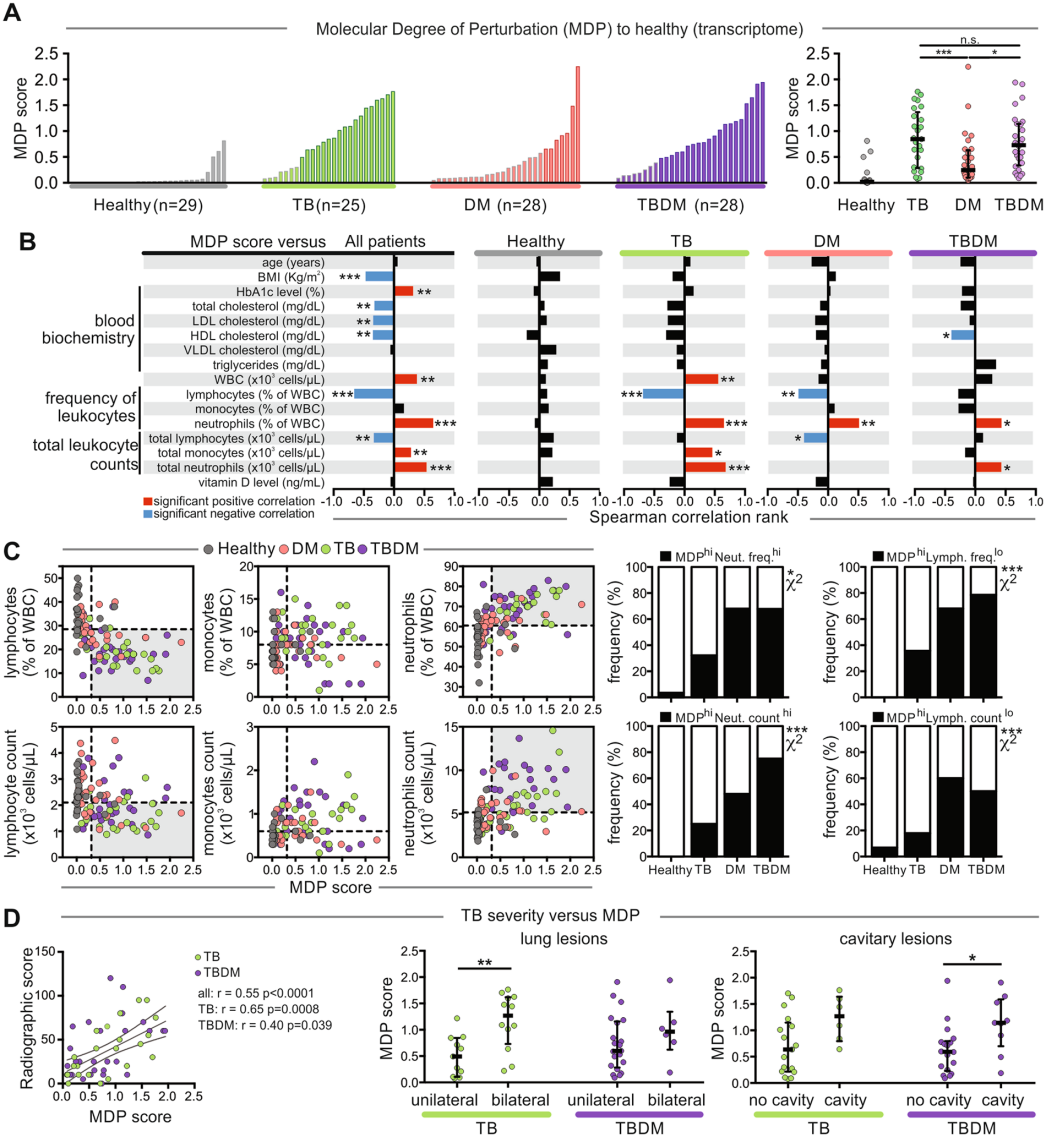
Factors associated with increased molecular degree of perturbation. (**A**) The molecular degree of perturbation (MDP) relative to healthy controls was calculated as described in Methods. Left panels show histograms of individuals in each subgroup. Right panel shows individual values with median and IQR per group. Data were compared using one-way ANOVA with Tukey’s multiple comparisons test, with single pooled variance. (**B**) MDP values were tested for correlations with the indicated parameters in each study subgroup using Spearman correlation ranks. Statistically significant correlations, after Holm-Bonferroni’s adjustment for multiple comparisons, are highlighted (red, positive correlation; blue, negative correlation; black, non-significant correlation). (**C**) Left panel shows correlation plots between differential WBC counts (neutrophils, monocytes and lymphocytes) and the MDP in all study groups. Dotted lines represent median values for MDP or leukocyte counts/frequency for the entire study population. Shaded areas indicate the individuals with the highest MDP values and the lowest lymphocyte counts/frequency or the highest neutrophil counts/frequency relative to the study population. Right panel shows comparisons of the frequencies in each study subgroup of individuals from the shaded areas on the left. Frequency comparisons were performed using the chi-squared test. (**D**) Left panel, MDP score was tested for correlation with the radiographic severity score (Spearman rank test). Right panel shows comparisons of MDP values in patients diverging in terms of TB disease distribution (unilateral vs. bilateral lung lesions) or presence of cavitary lesions. Within each study subgroup, values were compared using the Mann-Whitney U test. In all comparisons: *P < 0.05, **P < 0.01, ***P < 0.001.

The molecular distance to health (MDH) is a numerical score of transcriptional perturbation made by summing the differences in the expression levels of pre-selected genes between a given sample group and healthy control participants^31^. When applied to the blood transcriptome, MDH largely reflects immunological activation and it was reported to be higher in TB patients with more severe TB disease on chest x-ray^32^. To assess global effects of TB and DM on the blood transcriptome we quantified the molecular degree of perturbation (MDP) in the transcriptome of each patient. The MDP is adapted from the MDH and conveys similar information but has the advantages of controlling for variability in the control group and allowing for cross-dataset comparisons (see Materials and Methods). Median MDP vs. the healthy control transcriptome was elevated to a comparable degree in the TB and TBDM subgroups compared to participants with DM alone (Fig. 5A). Correlation of the expression values of each individual gene with MDP demonstrated a preponderance of downregulated transcripts in the DM subgroup vs. predominantly upregulated transcripts in TB patients with TBDM spanning these two extremes (Supplemental Figure 1). Across the total cohort, BMI, % and total lymphocytes, total cholesterol and all cholesterol subfractions were negatively correlated with MDP, while positive correlations were identified with HbA1c, total WBC, % and total neutrophils and % monocytes (Fig. 5B). Within the participant subgroups, a negative correlation of HDL cholesterol and MDP was identified only in TBDM patients, while neutrophils were positively correlated with MDP in the TB, DM and TBDM subgroups. Of note, the frequency of participants with high MDP and high absolute neutrophil count was greatest in the TBDM subgroup (Fig. 5C). These observations suggest an association of peripheral blood neutrophil numbers in with TB disease severity as reflected by MDP in patients with TB or TBDM. The data also suggest an association of low HDL cholesterol with the hyperinflammatory response of TBDM patients while high BMI was associated with a lower level of systemic inflammation in all subgroups.

The MDH was previously shown to correlate with the radiographic extent of TB disease estimated as mild, moderate or advanced using a widely adopted but non validated categorical scoring method^32^. In the current study, we graded chest x-ray severity using a numerical scoring method validated for smear-positive pulmonary TB^33^. This continuous radiographic severity variable correlated with MDP in TB and TBDM patients (Fig. 5D). Statistically significant correlation of MDP with the presence of bilateral vs. unilateral lung involvement was identified in TB patients, with a similar trend in TBDM. The presence of cavitary lesions correlated significantly with MDP in TBDM, with a similar trend in the TB subgroup. We conclude that immune activation as reflected by whole blood MDP is comparably elevated across the groups of TB and TBDM patients and correlates at the individual level with immune pathology and disease severity estimated by chest x-ray. These data further support the association of neutrophilic inflammation with TB severity, a relationship that is particularly strong in the TBDM subgroup.

### Classification of active TB by whole blood gene expression

**Figure 6:**
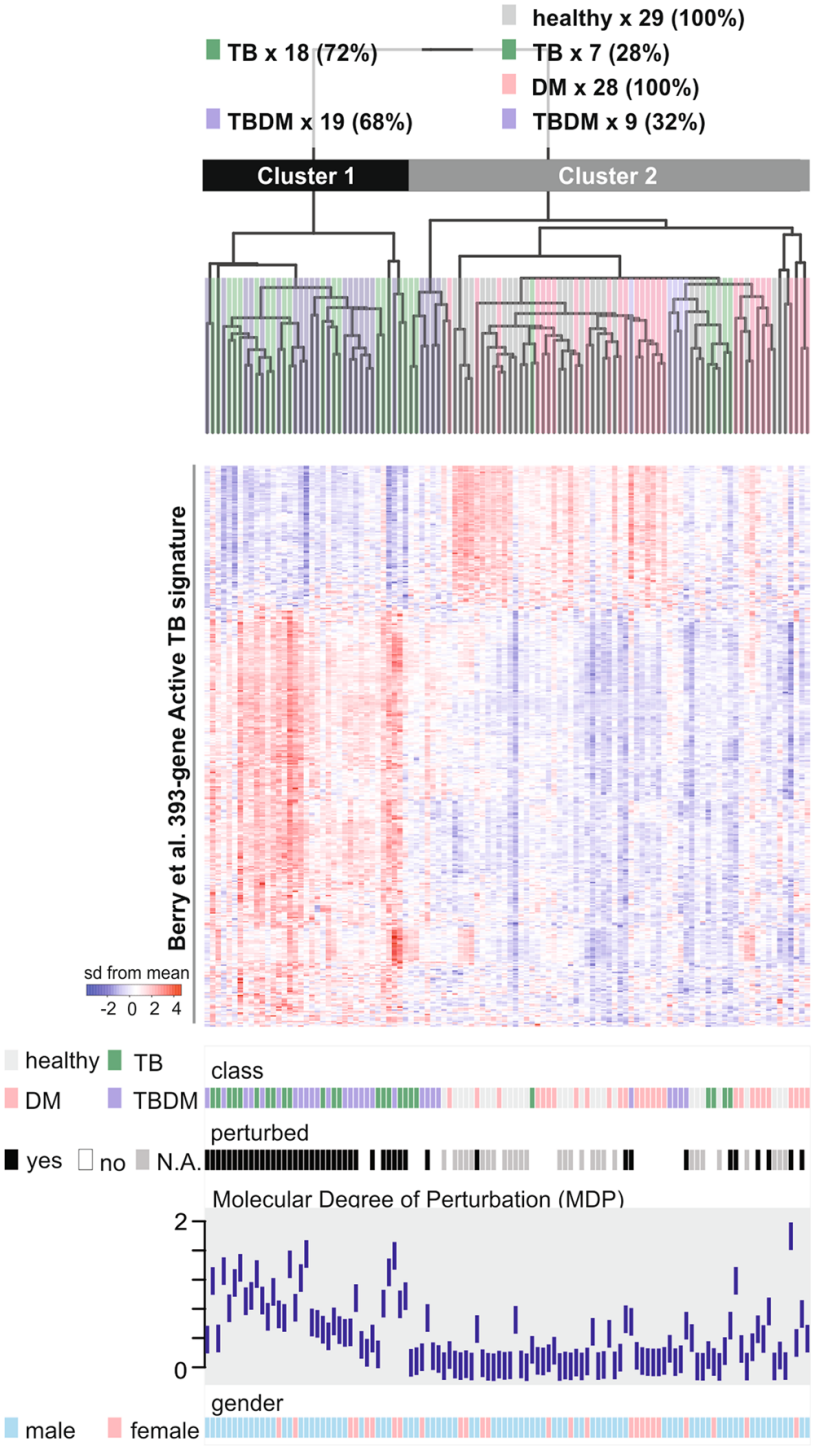
A blood transcriptional signature of active TB classifies most patients with or without diabetes. We applied a previously defined 393-gene signature of active TB^32^ to the 387 genes represented in the microarrays used for the present study. Expression profile of each patient and health subject (column) was ordered by hierarchical clustering (Spearman correlation with average linkage). The condition tree at the top shows 2 main clusters. The number of individuals from all each clinical subgroup (class) who segregated into each cluster is indicated at the top of the figure. Colored blocks at each profile base represent the different classes, molecular perturbation status, MDP score and gender.

Blood transcriptional signatures may inform the development of rapid diagnostic tests to distinguish between TB infection and TB disease. To determine whether comorbid DM alters the expression of TB signature genes, we analyzed the expression of a previously described 393-gene TB signature^32^ using the microarray dataset from our cohort, ordering the results by hierarchical clustering (Fig. 6). By this approach, 72% of nondiabetic participants with TB and 68% of participants with TBDM were correctly classified by the active TB disease signature. The second main cluster accurately classified 100% of healthy controls but misclassified 28% in the TB subgroup and 32% in the TBDM subgroup. The clustering results were compared at the individual level with MDP, which revealed that most patients with active TB who were misclassified in the healthy control cluster had lower MDP than the TB and TBDM subgroup members who were accurately classified as having active TB (Fig. 6A). Despite this moderate level of correct classification using the 393-gene signature, we noted that DEGs common to TB patients with or without DM in our cohort included all four genes from a 4-gene signature proposed by Maertzdorf *et al*. as the basis for developing a point-of-care diagnostic test^23^ (Fig. 3A). Another recently described 16-gene signature was shown to predict progression from TB infection to disease in independent South African and Gambian cohorts^24^. In our Indian cohort, DEGs in the nondiabetic TB subgroup included 11 DEGs present in the 16-gene signature, while 9 DEGs in the TBDM subgroup overlapped with the 16-gene signature (Fig. 3A). Conclusions are limited since we only had access to a single cohort of diabetic and nondiabetic South Indian TB patients. Additional studies will be needed to clarify the accuracy of TB classification based on blood transcriptome in South Indian populations with or without comorbid DM.

### Comorbid TB and DM amplify pathways associated with diabetic complications

**Figure 7:**
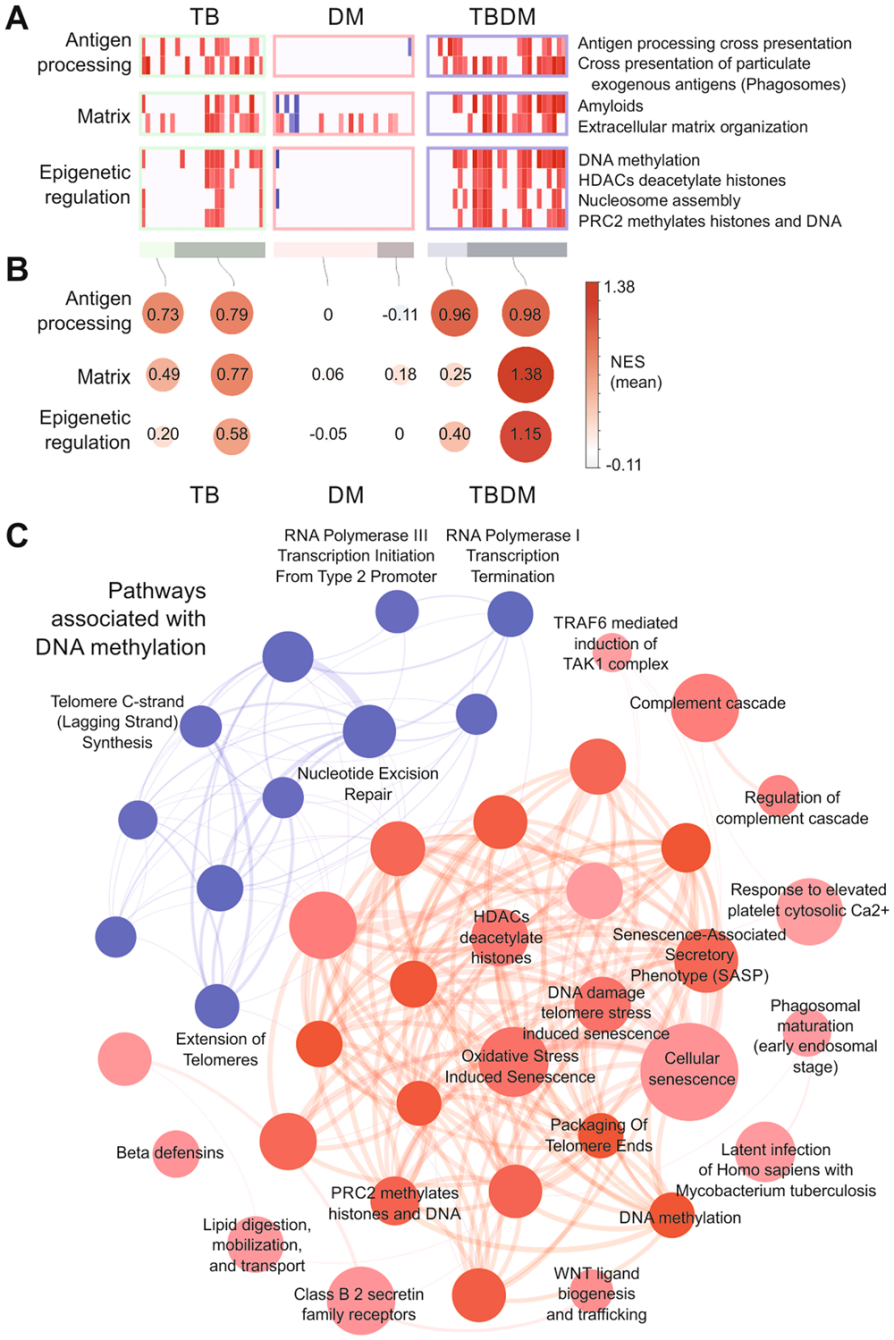
Increased activity of pathways associated with epigenetic regulation in diabetic TB patients. (**A**) Single sample Gene Set Enrichment Analysis (ssGSEA) was performed for each patient using the genes ranked by their MDP score and Reactome pathways as gene sets (*P* < 0.01, 1,000 permutations). Colors represent increase (red) or decrease (blue) in pathway expression activity vs. healthy controls as given by the normalized enrichment score (NES). Bars at the bottom indicate patients which are perturbed (darker color) or not-perturbed (light color). (**B**) Mean NES (values inside the circles) of the patients and pathways for each functional group. The size of the circles is proportional to the mean NES and color indicates positive (red) or negative (blue). (**C**) Pathways associated with “DNA methylation”. Spearman correlation between the expression activity of “DNA methylation” pathway and all other Reactome pathways across all patients. The network shows the pathways with correlation >0.6 (red circles) or <−0.6 (blue circles). The thickness of the edges is proportional to the number of genes in common between two given pathways (nodes).

To further explore blood transcriptomic perturbation uniquely associated with TBDM comorbidity, we performed single sample gene set enrichment analysis (ssGSEA) for individual participants using genes ranked by MDP score and Reactome pathways as gene sets (Fig. 7; *P* < 0.01, 1000 permutations). Additional information about the ssGSEA approach is presented in the README tab of Supplemental File 3. This analysis identified increased activity of several pathways linked to epigenetic regulation, which is increasingly understood to drive diabetic complications and explain the clinically recognized phenomenon of metabolic memory^34^. We then searched for pathways whose transcriptional activity was associated with the activity of “DNA methylation” pathway, and use the correlations among them to create a network (Fig. 7C). This analysis revealed an intricate association of DNA methylation with a broad range of pathways potentially related with the DM or TB pathogenesis, including “latent infection of *Homo sapiens* with *Mycobacterium tuberculosis*”, “TRAF6 mediated induction of TAK1 complex”, “Complement cascade”, “Lipid digestion, mobilization, and transport”, and “Cellular senescence” (Fig. 7C). Additional pathways linked to diabetic complications, including TRAF6, complement, platelet activation and RAGE receptor^35–38^ were also enriched, albeit to a lesser extent. Regardless of magnitude, the combined effects of the complication pathways activated in TBDM and linked to TB susceptibility suggest bidirectional pathological interactions between TB and DM.

### Integrative analysis reveals the potential drivers of the plasma cytokine changes in TBDM comorbidity

**Figure 8:**
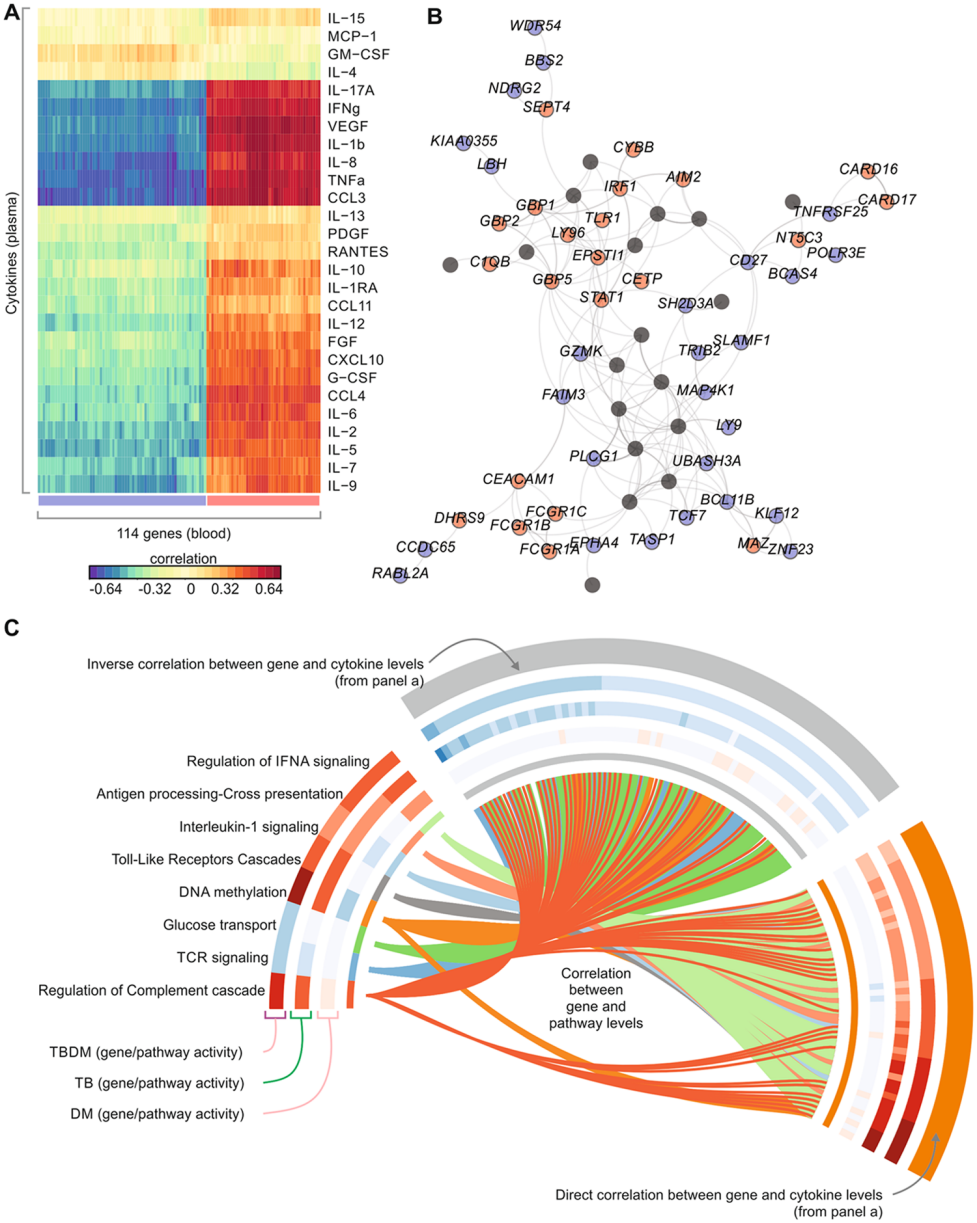
Integrative analysis of cytokines, gene expression and pathway activity in TBDM. (**A**) Integration of cytokine levels and microarray gene expression. Sparse group partial least square methods were used to identify the genes whose expression best correlates with most of the cytokines. Colors represent the correlation values between the expression of genes in blood (columns) and the levels of cytokines in plasma (rows). (**B**) Network containing some of the 114 genes from (A) GeneMania program was used to define the interactions (edges) between genes (nodes), and Gephi program to visualize the network. Colors represent is positive (red) or negative (blue) correlation of gene expression with the cytokines. (**C**) Integration of gene expression and pathway activity. Circos plot shows 8 selected pathways and 114 genes from (**A**). Each pathway was assigned to one color (inner circle). The lines connecting pathways and genes represent a high correlation (spearman correlation above 0.6) between the activity of the pathway (defined by the single-sample enrichment score) and the gene expression. Heat maps shown in the outer circles represent the activity of pathways (mean Normalized Enrichment Score) and genes (mean log_2_ fold-change in patients compared to healthy subjects). Colors indicate increase (red) or decrease (blue) in pathway activity or gene expression fold-change.

We applied the sparse partial least squares regression (sPLS) method^39^ to identify the minimum number of genes whose expression best explains most of the observed cytokine variation in TBDM (Fig. 8A). This approach identified 114 genes, many with key roles in innate and adaptive immunity (e.g. *BATF2*, *AIM2*, *CD27*, *FCGR1A*, *FCGR1B*, *FCGR1C*, *GZMK*) as well as histone genes (Fig. 8B, Supplemental File 4). Notably, plasma proteins associated with neutrophil recruitment and activation (IL-1b, IL-8, IL-17A, CCL3, TNFa, VEGF) were among those having strongest positive and negative correlations with gene expression values. Integrating the expression of these 114 genes with the activity of Reactome pathways, confirmed that most of the genes were associated with IFN, DNA methylation and antigen processing cross-presentation (Fig. 8C, Supplemental File 4). The patterns of gene/pathway activation were qualitatively similar for the TBDM and TB groups, and distinct from DM without TB (Fig. 8C). On a quantitative basis, the TBDM group had a greater increase in the activity of the “Regulation of complement cascade” and particularly the “DNA methylation” pathways as compared to TB alone. Taken together, these data suggest that epigenetic reprogramming and neutrophilic inflammation play a role in the signature pattern of plasma cytokines and growth factors in TBDM. While causation cannot be inferred from the available data, these may offer targets for therapeutic intervention.

## Discussion

The global population-attributable fraction of DM for adult TB cases (estimated to be 15% in 2013)^40^ exceeds the burden attributable to HIV and will continue to rise rapidly in countries with high TB burden and increasing prevalence of DM^41^. The dual burden of TB and DM is particularly acute in India, which has the largest number of TB cases (23% of global burden) and the second highest estimated number of DM cases (65.1 million) in the world^42^. There is an urgent need to understand the mechanisms and consequences of the interactions between DM and TB in order to define research priorities and provide a rational basis for the development of interventions. To address this gap in knowledge we collected clinical data, measured plasma cytokines and growth factors, and analyzed whole blood gene expression in a prospectively recruited cohort of South Indian adult pulmonary TB patients who were rigorously classified as diabetic or normoglycemic.

The immunological basis of diabetic TB susceptibility is not completely understood but insights have been gained from animal models^43^. Diabetic mice exhibit a critical delay in adaptive immune priming attributable to defective sentinel function of resident alveolar macrophages^3, 44^. Once underway, the T cell response in diabetic mice appears to be functionally intact but quantitatively excessive, possibly reflecting higher antigen load and/or defective counterregulation^4^. On this basis, we predicted that the immune response in TBDM patients would be qualitatively similar but quantitatively greater than that occurring the in the majority of nondiabetic TB patients.

Bayesian network analysis placed neutrophils at nexus of the connection between DM and TB (Fig. 1B). An association of neutrophilic inflammation with TB severity in the general population is well recognized^45^. Our data indicate that comorbid DM amplifies this response, which might reflect high bacterial load and/or a specific perturbation of immune function. Elevated of levels of plasma cytokines and growth factors distinguished participants with TBDM from two thirds of nondiabetic TB cases and all but one DM control whereas there was substantial overlap between the nondiabetic TB group and the diabetic controls without TB (Fig. 2). Results for the TBDM group are consistent with and extend published human and mouse data^2, 4, 17–19^ while the overlap between nondiabetic participants with TB and diabetic controls without TB was unexpected. The latter result points to a need for rigorous classification of glycemic status when interpreting proteomic data, at least in South Indian populations where DM appears to be a strong confounding factor.

The first published blood transcriptomic signature of active TB in cohorts from the United Kingdom and South Africa^32^ highlighted a role for type I IFN signaling in neutrophils. Fourteen subsequent transcriptomic studies analyzed gene expression in whole blood or isolated leukocyte fractions in TB patient cohorts from Africa, Europe, Indonesia and China^46^. Notably, none of these studies focused on TBDM comorbidity or included patients in India. Consistent with our prediction, the qualitative transcriptional response in TBDM closely matched that of the nondiabetic TB signature in terms of DEGs vs. healthy or diabetic participants without TB disease. An important conclusion is that comorbid DM does not appear to confound the clinical application of transcriptional profiling to TB diagnosis. Contrary to our expectation, there was no significant trend for higher MDP in TBDM vs. TB (Fig. 5A) although the TBDM subgroup had the highest proportion of individuals with combined high neutrophil count and high MDP (Fig. 5C). High MDP was positively correlated with radiographic severity, high neutrophil numbers and low BMI (Fig. 5B–D). The data are consistent with evidence that higher BMI is protective in TB^47^ but this association is lost in diabetic obesity, presumably as a consequence of diabetic immunopathy. An association of low HDL with high MDP was unique the TBDM subgroup. HDL cholesterol modulates innate and adaptive immunity through interactions with lipid rafts on myeloid and lymphoid cells^48^. The low HDL cholesterol associated with type 2 DM and pre-diabetes might be a glucose-independent factor contributing to TB susceptibility. We conclude that the transcriptional response to TB is qualitatively and quantitatively similar in the presence or absence of DM but confirmatory studies examining larger numbers of patients are warranted as the relatively small sample size and high degree of individual variation in MDP seen in all four subgroups were limiting factors.

While the blood transcriptional response did not distinguish TBDM from nondiabetic TB patients, 436 DEGs identified in the comparison of TBDM vs. DM were not shared in the comparison of nondiabetic TB to DM patients (Fig. 4). A finding with potentially high biological and clinical significance was that many of these “TBDM unique” DEGs participate in pathways that promote diabetic complications. Across a wide range of comparisons in this study, the amplification of genes, cytokines, growth factors and other biomarkers associated with diabetic complications was observed. These included but were not limited to the metrics of NLR and MHR, the plasma levels of VEGF, PDGF and FGF, and pathways associated with DNA damage and oxidative and ER stress, complement and platelet activation, RAGE signaling, and genes and pathways involved in epigenetic remodeling. We propose that a syndemic relationship exists between TB and DM that is conceptually similar to the relationship between TB and HIV^49^. Amplification of diabetic complication pathways during TB disease could be a factor in the increased early mortality of TBDM vs. DM^50^ and might exacerbate cardiovascular disease, kidney dysfunction, neuropathy and retinopathy in TB survivors. Conversely, the activation of pathways linked to beta cell loss could accelerate progression of dysglycemia. These questions are amenable to clinical investigation and TBDM patients might derive particular benefit from host-directed therapies targeting dyslipidemia, oxidative and ER stress, and other diabetic complication pathways such as RAGE signaling for which a robust drug development pipeline is already in place^51^.

Weaknesses of the present study include relatively small sample size, the high degree of individual variability in transcriptional response, heterogeneity within and between participant subgroups, and sample and data collection from the single time point of TB diagnosis. These deficiencies were partially compensated by the selection of a study site and patient population highly relevant to the TBDM syndemic. Results from EDOTS study screening indicate that 54% of adult pulmonary TB patients currently presenting to the government clinic system in Chennai are diabetic and another 21% are prediabetic^8^, making this a clinically important association regardless of causation. Additional strengths were the rigorous case definitions for glycemic status based on oral glucose tolerance test and fasting plasma glucose, the diversity of clinical parameters measured and the application of a validated radiographic scoring method.

In this systems biology investigation of TBDM comorbidity in a South Indian cohort we found no evidence for a discrete defect in the immune response to established infection. This was in keeping with studies from the mouse TBDM model that identified impaired kinetics of innate immunity following inhalation of *M*. *tuberculosis* and an associated delay in adaptive immune priming as key factors in diabetic TB susceptibility. These early event occurs before patients with active TB seek medical attention and can only be inferred in clinical research by subsequent effects disease evolution. No unique TBDM transcriptional signature was identified in this study; future studies in multiple, larger cohorts will be required to generate training and validation datasets to definitively address the question whether a discrete transcriptomic signature of TBDM exists. Despite limitations, the data presented here confirm the association of TBDM with increased, neutrophil-rich inflammation^52^. An unanticipated finding was that incident TB disease upregulated a broad range of genes and pathways involved in the progression of diabetic complications, above the levels seen in diabetic participants without TB. A particularly strong signal was seen for genes and pathways associated with epigenetic regulation (Figs 6 and 7). Epigenetic reprogramming could explain the persistence of DM-related phenotypes of T cells and macrophages adoptively transferred into non-diabetic recipients^4, 44^ and epigenomic investigation in peripheral blood leukocytes is promising direction for future clinical studies. Diabetic complication mechanisms, including epigenetic reprogramming, may contribute to the adverse effects of comorbid DM on TB outcomes and offer targets for therapeutic intervention in the rapidly growing number of people living with this dual burden of communicable and non-communicable diseases.

## Materials and Methods

### Study site and population

Participants with active pulmonary TB disease and either DM that preceded incident TB or normoglycemia were selected from those enrolled in the EDOTS cohort study in Chennai, India^8^. Briefly, adult smear-positive TB suspects from public clinics in Chennai were consented for screening if they conformed to the inclusion criterion of age 25–60 yr and lacked excluding criteria of treatment for prior episodes of TB disease, >7 d treatment for the current TB episode, >7 doses of a fluoroquinolone within 30 d of screening, pregnant or nursing, HIV seropositive or taking immunosuppressive drugs. Eligible candidates reporting a prior history of DM were confirmed by HbA1c ≥6.5%^53^ and current use anti-diabetic drug treatment. Those without DM history were screened by fasting plasma glucose and oral glucose tolerance test (75 g glucose challenge). Glycemic status based on plasma glucose 2 h post-challenge according to World Health Organization criteria^54^: DM (≥200 mg/dL) and normoglycemia (<140 mg/dL). Enrolled participants classified as diabetic or normoglycemic were withdrawn if the sputum culture obtained at enrollment was final negative for *Mycobacterium tuberculosis*. Candidates with no prior DM history but meeting the screening classification for DM were enrolled in the EDOTS cohort but were not included in the analysis reported here since they may represent a discrete subpopulation and could introduce unwanted heterogeneity. Sixty TB patients (30 with DM and 30 without DM) enrolled between January, 2014, and July, 2015, were selected from the EDOTS cohort for the current investigations. Selection was based on the closest age match between the TB and TBDM subgroups, with investigators blinded to other participant characteristics. A total of 60 control participants without TB (30 diabetic and 30 normoglycemic) were recruited from persons attending a DM screening clinic operated by the M.V. Hospital for Diabetes in the same urban location as the TB clinics. The absence of TB disease in control participants was confirmed by negative chest x-ray and sputum culture. Glycemic status classification in the control participants was confirmed by identical criteria as the TB subgroups.

### Laboratory Investigations

Complete blood counts, fasting lipid panel (total, low density lipoprotein [LDL] and high density lipoprotein [HDL] cholesterol, triglycerides) and 25-hydroxyvitamin D were measured for all participants in the laboratory of the M.V. Hospital for Diabetes. Chest x-rays were graded by two blinded readers using a validated radiographic TB severity scoring method^33^. All investigations reported here were conducted at the time of enrollment in the EDOTS cohort when participants had received <7 d of anti-TB treatment. Characteristics of the population are shown in Supplemental Table 1. Samples of plasma were purified and stored frozen at −80 °C prior to batch-wise Luminex assays (Bio-Rad, Hercules, CA). Samples of whole blood were stored at −80 °C in Tempus tubes (Applied Biosystems, Foster City, CA) prior to batch-wise preparation of total RNA using Tempus Spin RNA Isolation Kits (Thermo Fisher Scientific). Purified total RNA samples were delivered to Bionivid Technology Private Limited (Bangalore, India) for RNA quality control, labeling and hybridization on Illumina Human HT-12 v4 Expression BeadChips (Illumina Inc., San Diego, CA).

### Clinical and Laboratory data analyses

Median and interquartile ranges (IQR) were used as measures of central tendency. Frequencies were compared using the Pearson’s chi-squared test. Continuous variables were compared using the Mann-Whitney U test (between two groups) or the Kruskal-Wallis test with Dunn’s multiple comparisons (between >2 groups). Correlations were tested using the Spearman’s rank correlation test. The p-values were adjusted for multiple comparisons using the Holm-Bonferroni’s method^55^. Bayesian network learning was used to describe and visualize conditional dependencies between the multiple clinical and laboratorial variables. Continuous variables were discretized by Hartemink’s algorithm^56^ accessed by “bnlearn” package in R 3.1.0^57^. The learning algorithm used to establish the network structure was based on the heuristic Hill climb method^58^. The dependencies represented qualitatively by a directed acyclic graph where each node corresponds to a variable and a direct arc between nodes represents a direct influence. Robustness of the arcs was scored by a non-parametric bootstrap test (100 × replicates)^59^. Arcs with more than 30% support were depicted. The strongest associations were considered those remaining statistically significant in ≥60% bootstraps. All analyses were pre-specified. Two-sided *P* value < 0.05 after adjustment for multiple comparisons were considered statistically significant. Statistical analyses were performed using SPSS 20.0 (IBM statistics), Graphpad Prism 6.0 (GraphPad Software, San Diego, CA) and JMP 12.0 (SAS, Cary, NC, USA).

### Transcriptome analyses: Data acquisition and processing

RNA purity and integrity were evaluated by ND-1000 Spectrophotometer (NanoDrop, Wilmington, USA), Agilent 2100 Bioanalyzer (Agilent Technologies, Palo Alto, USA). Total RNA was amplified and purified using TargetAmp-Nano Labeling Kit for Illumina Expression BeadChip (EPICENTRE, Madison, USA) to yield biotinylated cRNA. Briefly, 300 ng of total RNA was reverse-transcribed to cDNA using a T7 oligo(dT) primer. Second-strand cDNA was synthesized, *in vitro* transcribed, and labeled with biotin-NTP. After purification, the cRNA was quantified on a ND-1000 Spectrophotometer (NanoDrop, Wilmington, USA). 750 ng of labeled cRNA samples were hybridized to each Human HT-12 v4.0 Expression Beadchip for 17 h at 58 °C, according to the manufacturer’s instructions (Illumina, Inc., San Diego, USA). Array signal detection was carried out using Amersham fluorolink streptavidin-Cy3 (GE Healthcare Bio-Sciences, Little Chalfont, UK) following the bead array manual. Arrays were scanned with an Illumina BeadArray Reader confocal scanner. Hybridization quality and chip performance were monitored by visual inspection of internal quality control checks and the raw scanned data. Raw data were extracted using Illumina GenomeStudio v2011.1 software (Gene Expression Module v1.9.0). Outlier samples were identified and removed using the arrayQualityMetrics R package^60^. Remaining samples per groups were: 29 Healthy, 25 TB, 28 DM and 28 TBDM. Probes whose expression value was not significantly above the detection limit (*P* < 0.05) in at least 50% of all samples were removed. Data were log2 transformed and corrected for batch effects using the ComBat algorithm implemented in the *sva* R package^61^. Probes matching the same gene symbol were collapsed by taking the one with highest expression across all samples. Mean and standard deviation for each gene using all samples were calculated and used to remove the 20% of the genes with lowest expression; and the 20% of the genes with lowest variance. The gene expression dataset used for subsequent analysis comprised 7,515 genes and 110 samples. Heat maps and “corrplots” were created using gplots, heatplus and corrplot R packages.

### Transcriptome analyses: Differentially expressed gene analysis

We defined 4 classes of samples: healthy, TB, DM and TBDM. Differential expression analyses were performed for each pair of classes using limma R package^62^. We considered DEGs as those genes having a limma adjusted *P*-value < 0.05 and an expression fold change >1.4. Ingenuity Pathway Analysis (Ingenuity Systems, Qiagen, Hilden, Germany) was used to identify canonical pathways and upstream regulators which were significantly enriched in the DEG lists (*P* < 0.01).

### Transcriptome analyses: Calculating the molecular degree of perturbation

The MDP method used for the present study is an adaptation of the MDH described by Pankla *et al*.^31^. Healthy controls were defined as the “reference” group, and the average expression level and standard deviation of this reference group were calculated for each gene. The MDP score of an individual gene (gMDP) in a given sample “s” was defined by taking the difference in expression level in sample “s” from the average of the gene in reference group divided by the corresponding standard deviation. Essentially, the gMDP score represents the number of standard deviations from reference. A filter was applied by replacing all absolute gMDP scores below 2 standard deviations by zero. Next, for each phenotype class (TB, DM and TBDM), we calculated the average gMDP score in the class, and used them to identify the top 25% genes with highest gMDP difference between the phenotype class and the reference group. Finally, the MDP of an individual sample (sMDP) was defined by taking the average absolute gMDP scores for all top 25% genes. This filter avoids absolute criteria while keeping the genes that best distinguish the control and test groups. To identify which sample was “perturbed”, we used a threshold consisting of the average sMDP scores plus 2 standard deviations of the reference group. Any sample above this threshold was considered “perturbed” (compared to our reference).

### Transcriptome analyses: Single-sample Gene Set Enrichment Analysis

Gene MDP scores were used to rank the genes for each participant. Gene Set Enrichment Analysis (GSEA)^63^ was run on these pre-ranked individual gene lists using the Reactome pathways^64^ as gene sets (GSEA, nominal *P* < 0.05; 1,000 permutations). GSEA calculated the normalized enrichment scores (NES) for the Reactome pathways based on the distribution of member genes of each module in the ranked list. We used the NES values to perform correlation analysis between two pathways or between genes and pathways. The network representing these correlations were created using Gephi.

### Transcriptome analyses: Whole blood 393-gene TB signature

The microarray platform used for the current study included 387 genes present in a previously reported 393-gene transcriptional signature of active TB (Berry *et al*.^32^). We used these we used these 387 genes as probes to cluster the samples in our cohort (Spearman correlation with average linkage).

### Integrative Analysis

Sparse partial least squares regression (sPLS) method implemented in the sgPLS R package^39^ was used to integrate gene expression and cytokine levels. Genes were used as ‘predictors’ and the cytokines as ‘respond variables’. This method decomposes both predictor and response variation while identifying their correlation and the contributors to each variation component. We determined the minimum number of genes (chosen among the most informative 300 genes) of the first predictor component that best maximize the explained-cytokine variation. In this way, we identified 114 genes which were then correlated (Pearson correlation) with the cytokine profiles allowing to identify the way in which the best explanatory genes correlate with cytokines. GeneMania^65^ was used to create an interaction network using as input the 114 genes. The information about “physical interactions”, “pathway”, “co-localization”, and “shared protein domains” was used to create the edges of the network. Gephi program was used to create the network. We then performed Spearman correlation between the expression of the 114 genes with the NES values (described in ‘Single-Sample Gene Set Enrichment Analysis’ section) of all Reactome pathways.

### Ethics Statement

This study was conducted according to the principles expressed in the Declaration of Helsinki and approved by the Ethics Committee of the M.V. Diabetes Research Centre (ECR/51/INST/TN/2013/MVDRC/01). Informed consent was obtained in writing from all participants.

## Electronic supplementary material

Supplementary Information

Supplementary Dataset 1

Supplementary Dataset 2

Supplementary Dataset 3

Supplementary Dataset 4
