# Supplementary material for: Systems Immunology of Diabetes-Tuberculosis Comorbidity Reveals Signatures of Disease Complications
Source: Sci Rep. 2017 May 17;7:1999. doi: 10.1038/s41598-017-01767-4 (PMC5435727; doi:10.1038/s41598-017-01767-4)
Supplement: Supplementary file 1 — Supplementary Information [file 41598_2017_1767_MOESM1_ESM.pdf]

Systems Immunology of Diabetes-Tuberculosis Comorbidity Reveals Signatures of Disease Complications

Cesar A. Prada-Medina, Kiyoshi F. Fukutani, Nathella Pavan Kumar, Leonardo Gil-Santana, Subash Babu, Flávio Lichtenstein, Kim West, Shanmugam Sivakumar, Pradeep A. Menon, Vijay Viswanathan, Bruno B. Andrade, Helder I. Nakaya, Hardy Kornfeld

Table S1. Characteristics of the study participants

Characteristic	Healthy	DM	TB	TBDM	P-value
**N**	30	30	30	30	
**Age - y (IQR)**	40 (31-48)	52 (45-61)	39 (31-52)	49 (39-54)	0.0002
**Male - no. (%)**	24 (80)	22 (73.3)	27 (90)	20 (66.7)	0.164
**BMI - Kg/m^2 (IQR)**	26.6 (23.2-28.5)	26.2 (22.4-28.1)	16.2 (15.5-19.6)	19.4 (15.8-22.7)	<0.0001
**Smoking status - no. (%)**					<0.0001
Current smoker	0 (0)	3 (10)	6 (20)	4 (13.3)	
Former smoker	1 (3.3)	1 (3.3)	13 (43.3)	6 (20)	
Never smoked	29 (96.7)	26 (86.7)	11 (36.7)	20 (66.7)	
**Alcoholism - no. (%)**					0.070
Current drinker	7 (23.3)	8 (26.7)	13 (43.3)	5 (16.7)	
Former drinker	4 (13.3)	3 (10)	8 (26.7)	7 (23.3)	
Never drank	19 (63.3)	19 (63.3)	9 (30)	18 (60)	
**Radiographic score**	-	-	30 (10-60)	25 (10-60)	0.790

Data were analyzed using the chi square (proportions) or the Kruskal-Wallis (continuous variables) tests, except for radiographic score values between TB and TBDM, which was analyzed using the Mann-Whitney *U* test. BMI, body mass index; IQR, interquartile range.

Table S2. Hematological characteristics of the study participants

Characteristic	Healthy	DM	TB	TBDM	P-value
**HbA1c - %**	5.5 (5.3-5.6)	7.5 (6.2-9.8)	5.7 (5.4-5.8)	10.2 (7.7-11.3)	<0.0001
**Vitamin D (ng/mL)**	15 (9.8-21)	18 (13.5-29.5)	16 (13-24.5)	12 (7-19)	0.030
**Total Cholesterol - mg/dL**	182 (156-211)	181 (161-213)	146 (127-164)	165 (137-206)	0.0003
**HDL Cholesterol - mg/dL**	43 (36-49.5)	41.5 (36.2-50.5)	37 (29-40.5)	35 (30-42.7)	0.003
**LDL Cholesterol - mg/dL**	99 (93-120.5)	98 (90.5-115.8)	79 (71-90.5)	97 (75-118.8)	<0.0001
**VLDL Cholesterol - mg/dL**	35 (23.5-42)	34.5 (25-47)	33 (22.5-40.5)	36.5 (29-47.7)	0.358
**Triglycerides - mg/dL**	88 (55.5-144)	109.5 (80.7-151.5)	74 (62-90.5)	73.8 (62.5-91.1)	0.0002
**WBC - 10^3/µL**	7.2 (5.7-8.3)	7.8 (6.4-10.1)	7.8 (6.9-9.9)	10.6 (8.8-13.0)	<0.0001
**Neutrophils - 10^3/µL**	3.8 (2.9-4.6)	5.2 (3.5-6.3)	5.2 (4.4-7.1)	7.8 (5.4-10.1)	<0.0001
**Monocytes - 10^3/µL**	0.5 (0.4-0.6)	0.5 (0.4-0.8)	0.8 (0.5-1.1)	0.9 (0.6-1.3)	0.0006
**Lymphocytes - 10^3/µL**	2.3 (2.1-2.7)	2.4 (1.7-2.9)	1.5 (1.2-2.0)	1.9 (1.6-2.5)	0.0002
**Monocyte: HDL ratio**	11.9 (8.3-24.7)	13.2 (9.3-20.6)	21.3 (14.4-32.7)	22.4 (11.6-39)	0.0005
**Neutrophil: Lymphocyte ratio**	1.7 (1.3-2)	2.2 (1.6-2.7)	3.7 (2.3-4.8)	4.1 (2.5-4.7)	<0.0001
**Monocyte: Lymphocyte ratio**	0.21 (0.16-0.27)	0.44 (0.30-0.84)	0.28 (0.18-0.35)	0.41 (0.29-0.65)	<0.0001

Data represent median and interquartile ranges. Values were compared using the Kruskal-Wallis. WBC, white blood cell count.

Table S3. Overall distribution of the parameters measured in serum.

Paramenter (Log_{10} pg/mL)	Healthy	TB	DM	TB & DM	P-value
**CCL2**	2.93 (2.83-2.99)	1.76 (1.19-2.39)	0.76 (0.20-1.64)	2.19 (1.65-2.50)	<0.0001
**CCL3**	0.77 (0.65-0.89)	1.51 (1.4-1.65)	0.66 (0.4-0.81)	2.01 (1.51-2.15)	<0.0001
**CCL4**	1.25 (0.92-1.54)	1.5 (1.32-1.87)	0.96 (0.65-1.23)	2.61 (2.36-2.82)	<0.0001
**CCL5**	4.79 (4.17-5.27)	4.79 (4.72-4.88)	5.34 (4.87-6.05)	6.18 (5.9-6.46)	<0.0001
**CCL11**	1.47 (1.29-1.57)	1.57 (1.18-1.71)	1.38 (1.21-1.63)	1.82 (1.62-2.1)	<0.0001
**CXCL10**	2.35 (1.99-2.49)	2.47 (2.29-2.67)	2.18 (1.90-2.44)	3.13 (3.04-3.37)	<0.0001
**FGF**	1.82 (1.61-1.89)	1.84 (1.61-1.89)	1.56 (1.23-1.88)	2.04 (1.92-2.26)	<0.0001
**G-CSF**	1.17 (0.83-1.28)	1.16 (0.98-1.38)	1.16 (0.92-1.32)	2.08 (1.82-2.2)	<0.0001
**GM-CSF**	1.73 (1.55-1.96)	0.97 (0.64-1.19)	1.15 (0.67-1.53)	1.73 (1.21-2.0)	<0.0001
**IFNγ**	1.92 (1.63-2.18)	2.41 (2.24-2.94)	2.01 (1.87-2.32)	2.92 (2.72-3.27)	<0.0001
**IL-1β**	1.11 (0.9-1.26)	1.93 (1.71-2.16)	1.52 (1.33-1.75)	2.34 (2.09-2.44)	<0.0001
**IL-1RA**	1.4 (1.32-1.45)	1.5 (1.36-1.66)	1.31 (1.16-1.57)	1.63 (1.57-1.74)	<0.0001
**IL-2**	1.56 (1.53-1.63)	1.82 (1.7-1.90)	1.83 (1.68-1.95)	2.21 (2.14-2.29)	<0.0001
**IL-4**	0.78 (0.63-1.01)	0.29 (0.10-0.75)	0.64 (0.43-0.77)	0.69 (0.42-1.06)	0.0003
**IL-5**	0.82 (0.59-1.04)	0.87 (0.77-1.3)	0.71 (0.38-0.95)	1.34 (1.09-1.87)	<0.0001
**IL-6**	1.70 (1.62-1.89)	1.85 (1.76-2.12)	1.87 (1.81-2.13)	2.32 (2.11-2.62)	<0.0001
**IL-7**	0.48 (-0.05-0.8)	1.32 (1.09-1.81)	1.22 (1.01-1.81)	1.57 (1.39-1.89)	<0.0001
**IL-8**	0.72 (0.57-0.89)	2.03 (1.91-2.2)	1.45 (1.37-1.65)	2.31 (2.17-2.54)	<0.0001
**IL-9**	0.84 (0.59-1.19)	1.89 (1.54-2.06)	1.3 (1.07-1.53)	1.8 (1.56-2.07)	<0.0001
**IL-10**	1.95 (1.93-1.97)	2.18 (1.87-2.38)	1.97 (1.6-2.19)	2.51 (2.2-2.59)	<0.0001
**IL-12**	1.6 (1.47-1.69)	2.15 (1.58-2.3)	1.78 (1.54-2.04)	2.36 (2.0-2.53)	<0.0001
**IL-13**	1.88 (1.29-2.36)	1.41 (1.0-1.81)	0.94 (0.16-1.3)	1.55 (1.46-1.61)	<0.0001
**IL-15**	2.07 (2.03-2.16)	1.43 (1.21-1.55)	1.07 (0.65-1.26)	1.73 (1.52-2.11)	<0.0001
**IL-17A**	1.76 (1.47-1.85)	1.94 (1.82-2.11)	1.81 (1.73-1.88)	2.32 (2.22-2.54)	<0.0001
**PDGF**	2.86 (2.56-3.05)	3.13 (2.66-4.33)	3.52 (2.73-4.21)	3.84 (3.41-4.21)	<0.0001
**TNFα**	1.45 (1.33-1.55)	2.3 (1.75-2.43)	1.64 (1.48-1.92)	2.6 (2.43-2.76)	<0.0001
**VEGF**	1.87 (1.59-2.22)	2.82 (2.71-3.07)	2.01 (1.85-2.38)	3.14 (2.82-3.44)	<0.0001

Data were compared the Kruskal-Wallis test. P-values were adjusted for multiple measurements as described in Methods.

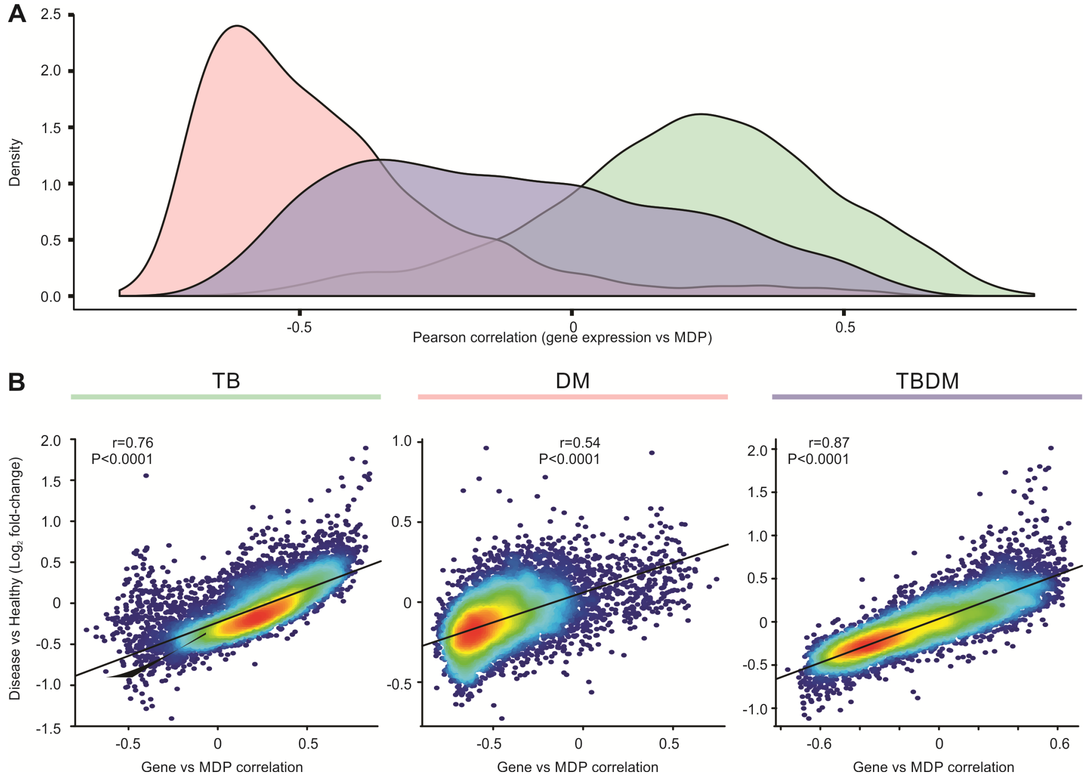

Supplemental Figure 1. Molecular degree of Perturbation (MDP) correlated to gene expression. (**A**) Distribution of R values for patients with TB (green), DM (pink) and TBDM (purple). Within each clinical phenotype, Pearson correlation was calculated between the MDP and expression values of each one of the genes. Kernel density plots were calculated in R. (**B**) Correlation between values in (A) and log2 fold-change between clinical phenotype and healthy subjects. Each dot represents one gene. Heat colors (from blue to red) represent the density of the dots (more overlap of dots, more red). Density Plots were generated in R.

Supplemental File Legends

File S1. Comparing the cytokine profiles of healthy subjects and TB, DM and TBDM patients.

Comparisons between the 4 groups were performed using limma R package. See "README" tab for details.

File S2. Differential expression gene and pathway enrichment analyses.

The "DEGs" tab shows the differentially expressed genes between each pair of classes (out of 4 in total: healthy subjects and TB, DM and TBDM patients). Comparisons between the 4 groups were performed using limma R package. The "IPA_upstream" tab shows all upstream regulators with p-value < 0.01 in at least one list of DEGs. The "IPA_CanonicalPathway" tab shows canonical pathways with p-value < 0.01 in at least one list of DEGs. See "README" tab for details.

File S3. Related and unrelated pathways linked to diabetic complications activated in patients with comorbid TB.

Single sample Gene Set Enrichment Analysis (ssGSEA) was performed for each patient using the genes ranked by their MDP score and Reactome pathways as gene sets (P < 0.01, 1,000 permutations). The "ssGSEA" tab shows the sum of Normalized Enrichment Score (NES) for all patients from each one of the 4 groups (healthy, TB, DM, TBDM). Only NES with nominal p-value < 0.01 were used. Reactome pathways with less than 15 genes were discarded. Reactome pathways not significantly enriched in any patient were discarded.

File S4. Integrative analysis applied to identify the potential drivers of the plasma cytokine changes in TBDM comorbidity.

The "Cytokine_vs_gene" tab shows the Pearson correlation matrix between cytokine levels (rows) and gene expression (columns). The "Pathway_vs_gene" tab shows the Spearman correlation matrix between Reactome pathway activity (rows) and gene expression (columns).
